# Boosting ART uptake and retention among HIV‐infected pregnant and breastfeeding women and their infants: the promise of innovative service delivery models

**DOI:** 10.1002/jia2.25053

**Published:** 2018-01-22

**Authors:** Meena Srivastava, David Sullivan, B. Ryan Phelps, Surbhi Modi, Laura N. Broyles

**Affiliations:** ^1^ Pediatric Maternal Clinical Branch Office of HIV/AIDS U.S. Agency for International Development Arlington VA USA; ^2^ Maternal and Child Health Branch Division of Global HIV & TB U.S. Centers for Disease Control and Prevention Atlanta GA USA

**Keywords:** HIV, differentiated care, service delivery models, antiretroviral treatment, ART, pregnant and breastfeeding women, HIV‐exposed infants, prevention of mother‐to‐child transmission of HIV, PMTCT

## Abstract

**Introduction:**

With the rapid scale‐up of antiretroviral treatment (ART) in the “Treat All” era, there has been increasing emphasis on using differentiated models of HIV service delivery. The gaps within the clinical cascade for mothers and their infants suggest that current service delivery models are not meeting families' needs and prompt re‐consideration of how services are provided. This article will explore considerations for differentiated care and encourage the ongoing increase of ART coverage through innovative strategies while also addressing the unique needs of mothers and infants.

**Discussion:**

Service delivery models should recognize that the timing of the mother's HIV diagnosis is a critical aspect of determining eligibility. Women newly diagnosed with HIV require a more intensive approach so that adequate counselling and monitoring of ART initiation and response can be provided. Women already on ART with evidence of virologic failure are also at high risk of transmitting HIV to their infants and require close follow‐up. However, women stable on ART with a suppressed viral load before conception have a very low likelihood of HIV transmission and thus are strong candidates for multi‐month ART dispensing, community‐based distribution of ART, adherence clubs, community adherence support groups and longer intervals between clinical visits. A number of other factors should be considered when defining eligibility of mothers and infants for differentiated care, including location of services, viral load monitoring and duration on ART. To provide differentiated care that is client‐centred and driven while encompassing a family‐based approach, it will be critical to engage mothers, families and communities in models that will optimize client satisfaction, retention in care and quality of services.

**Conclusions:**

Differentiated care for mothers and infants represents an opportunity to provide client‐centred care that reduces the burden on clients and health systems while improving the quality and uptake of services for families. However, with decreasing funding, stable HIV incidence, and aspirations for sustainability, it is critical to consider efficient, customized and cost‐effective models of care for these populations as we aspire to eliminate mother‐to‐child transmission of HIV.

## 1 Introduction

With the rapid scale‐up of antiretroviral treatment (ART) in the “Treat All” era, there has been increasing emphasis on using differentiated models of HIV service delivery. The intent of this differentiation is several‐fold: to improve client satisfaction and health outcomes, decrease the burden on congested health systems, reduce costs incurred by clients and health facilities, and develop more sustainable HIV programmes 1. The 2016 WHO Consolidated Guidelines on the Use of Antiretroviral Drugs for Treating and Preventing HIV Infection outline a basic approach to differentiated care in which HIV‐infected clients are categorized as “stable” (i.e. eligible for differentiated, often less intensive HIV services) or “unstable” (i.e. not eligible for differentiated service delivery models) 2. Within this proposed WHO framework, pregnant and breastfeeding women (PBFW) are classified as “unstable” and thus are not eligible for less intensive follow‐up given the concerns for mother‐to‐child transmission of HIV 2; this approach is also reflected in the exclusion of PBFW from recently published innovative service delivery interventions 3, 4. However, due to the recognition that PBFW can also benefit from differentiated service delivery approaches (especially PBFW stable on ART with suppressed viral loads), WHO convened a consultative meeting in late 2016 to assess current evidence for differentiated care models that include PBFW (and other key and vulnerable populations) 5. As a result, key considerations and a decision framework were released at the International AIDS Society Conference in 2017. This updated guidance provides potential criteria for inclusion of PBFW in differentiated care models with a particular focus on clinically stable PBFW who are on ART at conception 6, 7. Despite recent WHO guidance and the complexities of ante‐, peri‐ and postnatal care, a number of country programmes are reluctant to consider any PBFW for less intensive HIV service delivery models even as the international community seeks to find ways to offer less intensive HIV services to stable clients.

With the global expansion of “Option B+” for prevention of mother‐to‐child HIV transmission (PMTCT) and “Treat All” policies for people living with HIV, the number of women on ART in resource‐constrained settings has increased substantially; as a result, in many countries, at least half of HIV‐infected women presenting to antenatal (ANC) care know their HIV status and are stable on first‐line ART. Among these, the vast majority are virally suppressed 8. As a result of such PMTCT successes, new paediatric HIV infections have declined by 50% since 2010; however, the absolute number of newly HIV‐infected children remains unacceptably high 9. Data from PMTCT and maternal and child health programmes continue to reveal substantial programmatic challenges such as poor adherence to ART, high loss to follow‐up of post‐partum women, poor uptake of infant virologic testing and high mother‐to‐child transmission of HIV during breastfeeding 9, 10. The gaps within the PMTCT cascade for PBFW and their infants suggest that current service delivery models are not meeting families' needs and prompt a re‐consideration of how services are provided. This article will explore considerations for differentiated care for PBFW and their infants and encourage the ongoing increase of ART coverage through innovative strategies while still addressing the unique needs of this group.

## 2 Discussion

### 2.1 Factors to consider in defining stable PBFW and infants

In an effort to provide a more nuanced approach to differentiated care, the large umbrella term of “HIV‐infected pregnant and breastfeeding women” should be explored in greater detail. This dyad should not be grouped together and deemed inherently “unstable” in all contexts and circumstances. As the pregnancy and breastfeeding period can extend nearly three years in resource‐constrained settings, many women and infants will have prolonged periods of restricted access to differentiated care models with this approach, especially in areas of high fertility.

Service delivery models should recognize that the timing of the mother's HIV diagnosis is a critical aspect of determining eligibility for differentiated care. Women newly diagnosed with HIV during ANC, delivery or breastfeeding inherently require a more intensive approach so that adequate counselling and monitoring of ART initiation and response can be provided. Women on ART with evidence of virologic failure are also at high risk of transmitting HIV to their infants and require close follow‐up, especially since infants require enhanced prophylaxis per the 2016 WHO guidelines 2. However, women stable on ART with a suppressed viral load before conception have a very low likelihood of HIV transmission 11 and thus are strong candidates for multi‐month ART dispensing, community‐based distribution of ART, adherence clubs, community adherence support groups and longer intervals between HIV clinical visits 5.

A number of factors should be considered when defining eligibility of PBFW and infants for differentiated care. One consideration is the location of ANC and HIV services. Ideally, PBFW have access to an integrated, “one‐stop shop” model, but for women already on ART at pregnancy, providing ANC services in ART clinics is not the norm in resource‐constrained settings. HIV providers in ART clinics are rarely trained to provide routine obstetric care, so women are often referred to ANC for services, including HIV care and treatment. While this approach is still somewhat integrated, PBFW lose their “home” ART clinic and must temporarily re‐establish care in a different setting with a different provider. Although some PBFW may prefer this approach, it can be advantageous to keep stable PBFW on ART in their “home” clinic during pregnancy. It is recommended that if stable PBFW on ART are enrolled and choose to remain in a differentiated care model, they should be allowed to do so while still attending ANC services 6, 7. For those newly diagnosed with HIV, enrolling in differentiated care cannot be recommended until they meet basic criteria of “stability.” These considerations also apply to breastfeeding mothers and HIV‐exposed infants.

Another key factor in differentiated care for PBFW is access to HIV viral load monitoring. PBFW should be prioritized for viral load monitoring so that viraemia can be identified and acted upon quickly to prevent HIV transmission. By using viral load criteria to define stability, provider attention can be focused on PBFW with higher risk of transmission (as evidenced by an elevated viral load). In settings where viral load monitoring is not available, special consideration can be given to women with rising or stable CD4 per WHO guidelines, although such immunologic criteria are inferior and all efforts should be focused on providing the gold standard viral load monitoring 2.

WHO recommends at least 12 months on ART for non‐pregnant adults before being considered “stable” 2. Given the duration of pregnancy and breastfeeding, this is a reasonable approach for newly diagnosed PBFW and stable criteria should align with what has already been established by WHO for non‐pregnant adults living with HIV 6, 7. Upon delivery of the infant, every effort should be made to foster a “one‐stop shop” care approach to enable mother‐infant pairs to receive coordinated comprehensive care. For stable HIV‐infected breastfeeding women and their infants, a decreased frequency of clinic visits has the potential to improve the observed high loss to follow‐up in this population. However, as new rhythms of clinical care develop to improve retention, it is important to anticipate and avoid potentially detrimental impacts on the uptake of standard postnatal care, immunizations and timely infant virologic testing, especially as coverage of early infant testing and diagnosis at 6 to 8 weeks of age is inadequate in most countries in sub‐Saharan Africa. Models for breastfeeding women must therefore include components to track infants closely until a final seronegative status can be established or, for HIV‐positive infants, ART has been initiated.

### 2.2 Building on models that are working

Although there is encouraging anecdotal evidence supporting inclusion of PBFW in differentiated care, there is limited experience and data. In Cote d'Ivoire, integration of ANC and postnatal care services at the ART clinic for women who become pregnant while on ART resulted in improved post‐partum retention and reduced mother‐to‐child transmission of HIV at six weeks after birth 12. There are two existing models in South Africa that include post‐partum women and their HIV‐exposed infants. In the first model, a facility‐based educator enrolled high and low‐risk mother‐infant pairs at the postnatal 6 week visit to form postnatal clubs, which resulted in improved retention, viral load suppression of 98% for post‐partum women at six months, and zero seroconversions to HIV for infants 13. In the second model, post‐partum women and their HIV‐exposed infants participated in community‐based adherence clubs; this group had no differences in short‐term viral load outcomes compared to mother‐infant pairs referred to local primary healthcare clinics 14. Of note, one‐quarter of women in adherence clubs were not retained six months post‐partum, highlighting the need to ensure future studies not only include PBFW in differentiated care models but also evaluate transition between ART services in the post‐partum period 14.

To provide differentiated care that is client‐centred and driven, it will be critical to engage mothers and communities in models that will optimize client satisfaction, retention in care and quality of services. Each country context and setting will vary, and multiple models may be needed within communities to address the diverse needs of PBFW and their infants. When developing local solutions and models, input on these issues can be obtained by leveraging existing community and peer support networks such as lay counsellors, mentor mothers and mother‐to‐mother networks 15, 16, 17.

## 3 Conclusions

Differentiated care for PBFW and their infants represents an opportunity to provide client‐centred care that reduces the burden on clients and health systems while improving the quality of care and uptake of services. It is also important to recognize that differentiated care will allow healthcare providers to focus their time on mother‐infant pairs requiring more attention and support. Although the PMTCT cascade is complex, building sustainable long‐term models will provide a solid foundation for efforts to improve the health of mothers and families and eliminate mother‐to‐child transmission of HIV. PMTCT programmes will continue to expand as HIV testing and treatment become more widespread in the era of 90‐90‐90. However, with decreased funding, stable incidence, and aspirations for sustainability, it is critical to consider efficient, customized, and cost‐effective models of care for these populations as we aspire to eliminate maternal‐to‐child transmission of HIV.

## Competing interests

The authors declare that they have no competing interests.

## Authors' contributions

BRP and SM conceived the topic for the commentary. MS, DS and LNB developed the initial outline and draft. All authors reviewed and provided revisions to the draft. MS incorporated revisions and coordinated approval from all authors prior to submission.

## Funding

This publication was supported by the U.S. President's Emergency Plan for AIDS Relief through the U.S. Agency for International Development and the U.S. Centers for Disease Control and Prevention.

## Disclaimer

The findings and conclusions in this report are those of the authors and do not necessarily represent the official position of participating federal agencies, including the U.S. Agency for International Development and the U.S. Centers for Disease Control and Prevention.
